# Impact of clearcutting on radiocesium export from a Japanese forested catchment following the Fukushima nuclear accident

**DOI:** 10.1371/journal.pone.0212348

**Published:** 2019-02-14

**Authors:** Tatsuhiro Nishikiori, Seiji Hayashi, Mirai Watanabe, Tetsuo Yasutaka

**Affiliations:** 1 Center for Regional Environment Research, National Institute for Environmental Studies, Onogawa, Tsukuba, Ibaraki, Japan; 2 Fukushima Prefectural Centre for Environmental Creation, Fukasaku, Miharu, Fukushima, Japan; 3 Fukushima Branch, National Institute for Environmental Studies, Fukasaku, Miharu, Fukushima, Japan; 4 National Institute of Advanced Industrial Science and Technology, Higashi, Tsukuba, Ibaraki, Japan; University of South Carolina, UNITED STATES

## Abstract

Changes in ^137^Cs export over time following clearcutting were investigated in a Japanese forested catchment affected by the Fukushima nuclear accident. A total of 13% of the catchment area was clear-cut 2 years after the accident. Annual suspended solids (SS) export at the catchment outlet increased 1.4 to 2.0 times after clearcutting; however, ^137^Cs export increased slightly (up to 1.1 times), corresponding to 0.21% to 0.30% of the ^137^Cs inventory in the catchment. The smaller change in ^137^Cs export than in SS export was due to a rapid decrease in the activity concentration following clearcutting. This decrease was likely caused by both natural attenuation and SS derived from sources with a low activity concentration in the clear-cut area. Monitoring of the sediment transport from hillslopes in small-scale experimental plots showed that the ^137^Cs yield in the skid trail was 3.6 to 21 times greater than those in clear-cut and unlogged forest floors. This significant ^137^Cs transport was due to greater soil erosion (by up to two orders of magnitude) along the skid trail, despite the lower activity concentration than those in the other plots. This indicates that while skid trails were involved in the rapid decrease of the activity concentration of SS, they were a potential source of the increased export of ^137^Cs and SS. Net ^137^Cs export increased by clearcutting (the export excluding the decrease accompanied by natural attenuation) was estimated to account for only 0.092% of the inventory in the catchment for 2.5 years. These results imply that the impact of clearcutting on ^137^Cs export was temporary in this catchment.

## Introduction

The Great East Japan Earthquake and the following tsunami damaged the Fukushima Daiichi Nuclear Power Plant (FDNPP) on March 11, 2011 and resulted in the emission of a large amount of ^137^Cs (8.8–50 PBq) to the atmosphere [1], thereby contaminating a wide area (mainly forest) of eastern Japan. After the Chernobyl nuclear accident, forests in several European countries were contaminated with ^137^Cs, which persisted for a long time due to the long half-life (30.2 years) of the radioisotope and limited removal from the forest ecosystems [2,3]. A similar situation may have occurred in Japan, and many researchers have begun to investigate the state of contamination immediately after the accident.

In forests, the fallout ^137^Cs was partly trapped by tree canopies [4–7] and subsequently transferred to the forest floor via throughfall, stemflow, and litterfall [8–11]. At 2 years after the accident, more than 75% of the deposited ^137^Cs accumulated in the forest floor [12]. ^137^Cs in the forest floor has been retained in the top soil [13–16] due to strong adsorption/fixation by clay minerals [3] and assimilation by fungal mycelium [17,18]. This strong association leads to ^137^Cs mobilization via soil erosion. ^137^Cs transport on the forested hillslopes, however, was less than 1.1% of the initial inventory for 5 months to 1 year [19–21], and annual ^137^Cs export via streams was less than 0.3% of the total inventory in the forested catchments [22,23]. These results suggest that ^137^Cs removal from forest ecosystems is limited in Japan.

However, significant artificial disturbances, such as clearcutting, may result in large soil losses and sediment export. This leads to an increase in ^137^Cs export, which raises the contamination level in downstream areas, including floodplains, reservoirs, and irrigated farmlands [3,24,25]. Most contaminated forests have not been decontaminated [26]. Clearcutting thus might enhance the extra contamination in areas downstream of the forests.

Numerous studies have reported an increase in sediment export following clearcutting (e.g., [27–29]), and the increase varies tremendously among sites. Bathurst and Iroumé [30] reported an increase of 0.2–150 times in logged temperate forests worldwide. Similar variability has been reported for trace element export (e.g., mercury [31]). This suggests that clearcutting may also increase ^137^Cs export several-fold. For radiocesium, Wallbrink et al. [32] have reported that 97 ± 10% of ^137^Cs derived from nuclear weapons testing remained in a clear-cut catchment in Australia 6 years after clearcutting; nevertheless, there are very few such case studies. Consequently, more case studies are necessary to determine the impact of clearcutting on ^137^Cs export.

In Japan, more than 70% of the timber volume is stored in private forests [33], and 80% of the private forest owners each held areas of less than 10 ha [34]. This means that in many cases, only part of a catchment will be clear-cut. The impact of clearcutting on ^137^Cs export should be assessed in partially clear-cut catchments accordingly.

We investigated ^137^Cs export via suspended solids (SS) from a forested catchment in which 13% of the catchment was clear-cut 2 years after the Fukushima nuclear accident. To elucidate temporal trends, a shift in SS export patterns after clearcutting was statically examined, and ^137^Cs export was then compared between pre- and post-clearcutting periods. In addition, we compared ^137^Cs and sediment transport on disturbed and undisturbed hillslopes using small-scale soil erosion plots to reveal potential sources of increased ^137^Cs in streams. The plot sites included a skid trail, which is an important sediment source according to numerous studies (e.g., [35–37]). We discussed the mechanisms underlying changes in ^137^Cs export after clearcutting on the basis of these results.

## Materials and methods

### Site description

**Fig 1:**
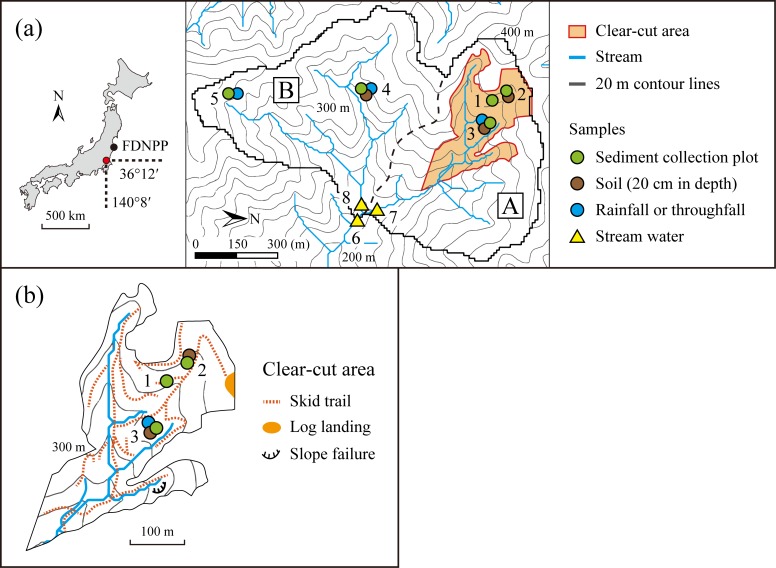
Study site and sampling locations. (a) Entire catchment. (b) Clear-cut area. 1, skid trail plot; 2, clear-cut cedar forest plot; 3, clear-cut cypress forest plot; 4, cedar forest plot; 5, cypress forest plot; 6, entire catchment outlet; 7, sub-catchment A outlet; 8, sub-catchment B outlet.

The forested catchment is located on Mount Tsukuba, 160 km southwest of FDNPP (Ishioka, Ibaraki, Japan; N36°11′56″, E140°7′52″) (Fig 1). Permission was granted for the field study by the Kanto regional forest office. The mean deposition density of ^137^Cs was calculated to be 13.4 kBq m^−2^ using airborne monitoring survey data [38]. The drainage area, altitude, and mean slope gradient of the catchment were 67.5 ha, 200–380 m, and 15°, respectively. At AMeDAS Tsukuba station, located 16 km south of the study site, the mean annual temperature and precipitation in 2006–2015 were 14.5°C and 1400 mm, respectively [39]. Monthly precipitation exceeded 100 mm in most months from April to October. Until clearcutting, 75% of the catchment area was covered with conifer plantations of Japanese cedar (*Cryptomeria japonica*) and Japanese cypress (*Chamaecyparis obtusa*) that were 23 to 55 years old, and the remaining 25% of the catchment was covered with 20-year-old broad-leaved forest with more than ten species of trees and lianas [6]. The rock in the catchment comprises granodiorite of the Paleocene to early Eocene and pelitic gneiss of the Cretaceous [40]. The stream bed consists of sand and gravel. The main soil type is classified as brown forest soil [41], which corresponds to Cambisols [42]. The surface soil consists of a litter layer (L horizon), fermentation and humus layer (F/H horizons), and top layer of mineral soil (A horizon).

The catchment is made up of sub-catchments A and B (Fig 1) accounting for 47% and 53% of the entire area, respectively. The upstream area in sub-catchment A was covered with cedar and cypress forests that were 55 years old. A total of 13% of the entire catchment area (8.8 ha) was clear-cut between November 2012 and March 2013 in the upstream area. During the clearcutting, unsealed skid trails were constructed by cutting and filling slopes using excavators. Trees were cut using feller bunchers and chain saws, and then the tree trunks alone were harvested and taken to a log landing on a ridge using forwarders. The residual leaves and branches were left on the forest floor or stacked with the logs in rows in the clear-cut areas as barriers for soil erosion prevention. The total area of the skid trails was approximately 0.6 ha, accounting for 6% of the clear-cut area. There were road-stream crossings at five points, two of which were constructed with culverts. At the rest, skid trails were directly connected to streams. A minor slope failure occurred beside a stream in the northern part of the clear-cut area within 1 year and 9 months after clearcutting (Fig 1). The volume was several cubic meters, and the sediment weighed several tons. Cedar and cypress seedlings were planted 1 year after clearcutting.

### ^137^Cs in sediment transferred on the hillslopes

We investigated ^137^Cs in sediment transferred on disturbed and undisturbed hillslopes: skid trail, clear-cut cedar forest, clear-cut cypress forest, unlogged cedar forest, and unlogged cypress forest (Fig 1). General information about these experimental sites is summarized in Table 1.

**Table 1:** General information about experimental sites for ^137^Cs transfer on hillslopes.

Site	Slope gradient (°)	Stand age (yr)	Stand density (ha^−1^)	Stand height (m)^a^	DBH (cm)^a^	Vegetation cover (%)^b^			Soil horizon thickness (cm)^c^		
						Canopy layer	Shrub layer	Herb layer	L horizon	F/H horizons	A horizon
**Clear-cut area**											
Skid trail	9					0	0	0			
Clear-cut cedar forest	27					0	0	5	1.0	1.0	>20
Clear-cut cypress forest	27					0	0	<5	0.5	3.5	>20
**Forested area**											
Cedar forest	27	53	1600	22 ± 2	25 ± 5	90	25	90	3.0	3.0	20
Cypress forest	27	24	2400	14 ± 0.6	16 ± 4	95	5	60	0.5	2.0	10

DBH, diameter at breast height.

Experimental sites were 225–400 m^2^.

^a^Mean ± standard deviation.

^b^Measurement date: May 2013 in the clear-cut area and September 2014 in the forested area.

^c^Measurement date: March 2016 in the clear-cut cedar and cypress forest and September 2014 in the forested area.

**Fig 2:**
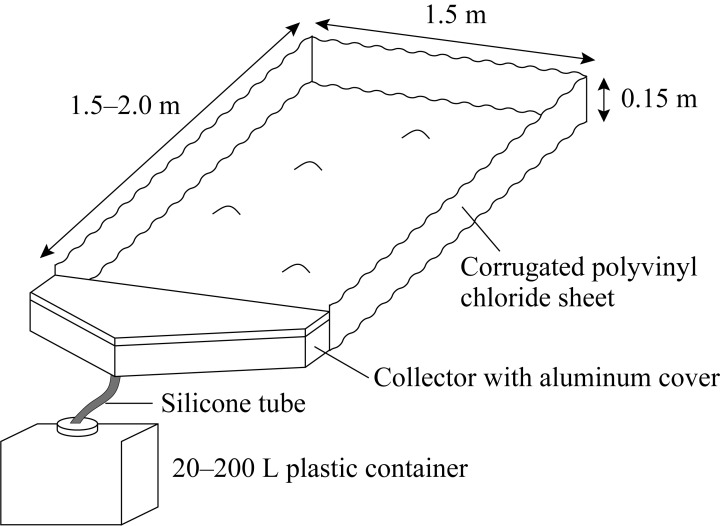
Schematic diagram of the sediment collection plot [19].

Experimental plots of 2.3 to 3.0 m^2^ were established for collecting sediment with runoff water using the method of Nishikiori et al. [19] (Fig 2). The plots were installed at points that were covered normally with understory vegetation and organic horizons. The slope gradients were the same as those presented in Table 1. The plots were located more than 5 m horizontally and more than 2 m high from streams.

The experiment began 1.5 months after clearcutting for 180 days (May 17 to November 13, 2013, spring to autumn; S1 Fig). Samples were collected monthly or after rainfall events with a total precipitation of several tens of millimeters. Precipitation during the sampling period was 890 mm, which accounted for 69% of the annual total according to observations 6 km northeast of the study site (AMeDAS Kakioka station) [39]. During the same period as the sediment sampling, precipitation was collected monthly in a sampler consisting of a polyethylene funnel (10 cm in diameter) and a polyethylene bottle (3 L) [6] at each site (Fig 1) as follows: bulk precipitation in the clear-cut area and throughfall in the unlogged forests of cedar and cypress.

Sediment was separated from runoff water samples by the following method. Runoff water samples were passed through a non-woven fabric cartridge filter to trap sediment (pore size of 1 μm, RP13-011, Japan Vilene Co., Ltd., Tokyo, Japan) [43]. The cartridge filters were dried at 105°C and weighed before and after runoff water passed through to determine the weight of the trapped sediment. The filters were placed in plastic containers (62 mm i.d. and 54 mm H; Fujiwara Scientific Co., Ltd., Tokyo, Japan) for a ^137^Cs radioactivity analysis. The filter has a limited capacity and cannot trap more than a few grams of sediment. For samples with high sediment content, the runoff water was sieved to 63 μμm and then passed through the filter. The sediment trapped on the sieve was dried at 105°C, weighed, homogenized, and packed in 100-mL plastic containers (50 mm i.d. and 62 mm H, U-8; As-one Co., Ltd., Osaka, Japan) for a ^137^Cs radioactivity analysis. The ^137^Cs activity concentration in samples with high sediment content was calculated by dividing the total activity of sediment trapped on the sieve and in the filter by the total sediment weight.

To determine ^137^Cs inventories in the plots, soil cores (surface area, 7.4 cm^2^) including organic horizons were taken to a depth of 10 cm at the outer four corners of the plots (i.e., n = 4) just before the beginning of the sediment collection experiment. ^137^Cs yields from the plots were calculated by multiplying the sediment yield and the activity concentration. Percentages of ^137^Cs transported were obtained by dividing the ^137^Cs yields by the inventory. To measure the vertical distribution of ^137^Cs, total C and N, and particle size distribution, soil cores with a depth of 20 cm (same surface area) were also collected in triplicate at the clear-cut cedar forest, clear-cut cypress forest, and unlogged cedar forest in March 2015. The cores for the vertical distribution were divided into 2- to 4-cm depth sections. All soil samples were dried at 105°C, weighed, and packed in the 100-mL plastic containers for the ^137^Cs radioactivity analysis.

After the ^137^Cs radioactivity analysis, soil samples for the particle size distribution analysis were resuspended with dispersant (sodium hexametaphosphate) and ultrasonic oscillation for 10 min immediately before obtaining measurements and then analyzed, covering the range from 0.05 to 3,000 μm, with a laser diffraction particle size analyzer (SALD-3100; Shimadzu Co., Kyoto, Japan). Soil samples for the analysis of the total contents of C and N were ground with an agate mortar and then analyzed using a CN analyzer (Sumigraph NC-Trinity, Sumika Chemical Analysis Service, Ltd., Tokyo, Japan).

### ^137^Cs export from the catchment

We investigated ^137^Cs export (flux) with SS at the catchment outlet (Fig 1) during and after clearcutting. During rainfall events, stream water was collected at the outlets of the entire catchment and of sub-catchments A and B (Fig 1). Samples were collected directly into polypropylene bottles or using an automatic water sampler (ISCO3700; Teledyne Technologies Inc., Thousand Oaks, CA) at intervals of 30 to 60 min. Sampling volumes were 1 to 6 L, and 1 to 7 samples were collected for each rainfall event. The numbers of runoff events sampled were 14, 8, and 4 for the entire catchment, sub-catchment A, and sub-catchment B, respectively.

Samples were homogenized by shaking the sampling bottles and were then divided into aliquots for analyses of the SS concentration, particle size distribution, and ^137^Cs activity. The SS concentration was measured by a filtering method using glass fiber filter papers (pore size, 0.7 μm, GF/F; GE Healthcare, Tokyo, Japan). Samples for the particle size distribution analysis were resuspended by ultrasonic oscillation for 30 min immediately before measurements and then analyzed following the measurement method described above. Samples for the ^137^Cs analysis were centrifuged at 15800 × *g* for 30 min with a high-speed centrifugal separator (Model 7000; KUBOTA Co., Tokyo, Japan), and then SS separated by centrifugation was placed in 100-mL plastic containers, dried at 60°C, weighed, and analyzed for ^137^Cs.

^137^Cs export via SS from the entire catchment was examined during the clearcutting and post-clearcutting periods from November 2012 to September 2015 (S1 Fig). Volumetric flow rate and turbidity were monitored at 10-min intervals using an area velocity Doppler flowmeter (MS-P2; PenTough Co., Ltd., Osaka, Japan) and a turbidity meter (INFINITY-Turbi; JFE Advantech Co., Ltd., Tokyo, Japan), respectively. The instruments were installed in the drainage canal at the outlet. Turbidity (FTU) was converted to an SS concentration (mg L^−1^) using the following relational expression: SS concentration = 1.98*FTU* − 2.22 (*R*^2^ = 0.97). Then, SS flux was calculated by multiplying the converted SS concentration and the volumetric flow rate. Finally, ^137^Cs flux was obtained by multiplying the SS flux and the ^137^Cs activity concentration in SS. These fluxes and related data during the pre-clearcutting period (May 2010 to October 2012) were obtained from Hayashi et al. [unpublished data], who calculated SS flux using the following LQ equation for the relationship between load [L: SS flux (g s^−1^)] and volumetric flow rate [Q (m^3^ s^−1^)]: *L* = 10^3.90^*Q*^2.57^ (*R*^2^ = 0.83). Turbidity monitoring began in March 2013, and the LQ equation was used to obtain SS flux from November 2012 to February 2013 (within the clearcutting period).

### Radiocesium analysis

Activity of ^137^Cs in samples was measured using a coaxial or a well-type germanium detector (GC2518 or GCW7023; Canberra, Meriden, CT). Spectrum Explorer (Canberra Japan, Tokyo, Japan) was used to analyze the γ-ray spectra. MX033U8PP (The Japan Radioisotope Association, Tokyo, Japan) was used as a standard source for the efficiency calibration. Estimates of ^137^Cs activity of samples trapped in the cartridge filters were corrected using the formula of Tsuji et al. [44]. ^137^Cs activity was decay-corrected to the sampling date, and the activity concentration (Bq kg^−1^ or kBq kg^−1^) was determined on a dry weight basis.

### Fitting a model to ^137^Cs activity concentrations

We used a nonlinear mixed effects model to fit the time-series ^137^Cs activity concentration in the SS as follows:

$$C_{i}=C_{0}{}_{i}\;\exp\left(-kD\right)+\varepsilon_{i}$$ (1)

where *C* is the ^137^Cs activity concentration (Bq kg^−1^), *C*_0_ is the activity concentration on the day following the completion of clearcutting, *k* is the empirically determined decrease rate constant of the activity concentration, *D* is the number of days after clearcutting, *ε* is an error term, and the subscript *i* indicates the replication. *C*_0_ is the random effect parameter for the dispersion of the repeated samples on each sampling day. R software [45] and the R package ‘nlme’ [46] were used to fit the nonlinear mixed effects model to the data. The activity concentration calculated from the model was used in the ^137^Cs flux calculation.

### Calculation of rainfall kinetic energy

Rainfall conditions affect the amounts of SS and ^137^Cs export. Rainfall kinetic energy, which is a factor in soil erosion models, such as USLE [47], was calculated to evaluate the relationship with the amount of SS export. Rainfall kinetic energy is given by the following equation [48]:

$$e_{K}=0.028\left[1-0.52exp\left(-0.042R\right)\right]$$ (2)

$$E_{K}=e_{K}R$$ (3)

where *e*_K_ is rainfall kinetic energy per unit rainfall depth (GJ km^−2^ mm^−1^), *R* is rainfall intensity (mm h^−1^), and *E*_K_ is rainfall kinetic energy per unit time (GJ km^−2^ h^−1^). The rainfall intensity recorded at 60-min intervals was obtained from the AMeDAS Kakioka station located 6 km northeast of the study site [39]. The rainfall kinetic energy during the investigation period was calculated by integrating *E*_K_ values.

Regression lines for monthly rainfall kinetic energy and monthly amount of SS export were derived for pre- and post-clearcutting periods, and the homogeneity of the regression coefficients was examined by testing the interaction effect in analysis of covariance. A similar test was performed for LQ equations in pre- and post-clearcutting periods.

## Results

### ^137^Cs transport from hillslopes

**Fig 3:**
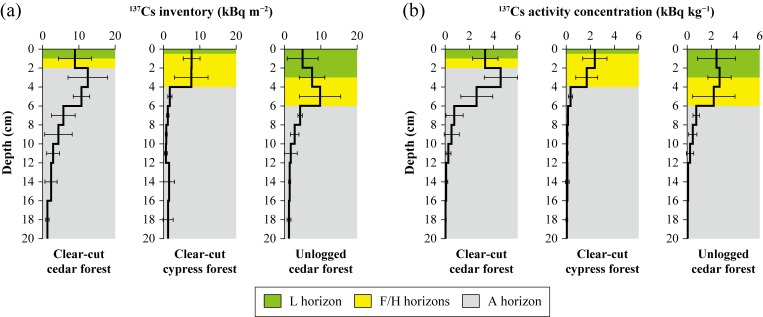
**(a) Depth profiles of mean inventories and (b) mean activity concentrations of** ^**137**^**Cs in clear-cut and unlogged forest sites.** Error bars represent the standard deviation for three samples.

For the ^137^Cs vertical distributions in soil, the amounts retained in the upper 6-cm soil horizon were 66 ± 15% and 77 ± 12% (means ± standard deviations) of the total inventories in clear-cut cedar and cypress forests, respectively, even 2 years after clearcutting (Fig 3A). Similarly, in unlogged cedar forest, the upper 6-cm horizon trapped 62 ± 18% of the total inventory. The activity concentrations were also high in the upper 4- or 6-cm soil horizon (Fig 3B). These results indicate that ^137^Cs was retained in the surface soil horizon, most vulnerable to erosion, throughout the study period. Detailed data, including physical properties and total contents of C and N, are presented in S1 Table.

In the experimental plots, the inventories and activity concentrations in soil did not differ significantly between the plots based on the Steel-Dwass test (data were heteroscedastic) (Table 2), although the skid trail had an order of magnitude lower values than those of the other plots. Bulk density differed significantly between plots (*p* < 0.01) based on Tukey’s test (data were homoscedastic), and the skid trail had a bulk density that was over two times higher than those in the other plots.

The maximum sediment yield was observed in the skid trail and was one to two orders of magnitude larger than those for the other plots (Table 2). The second largest value was observed in the clear-cut cypress forest and was an order of magnitude larger than those in the other plots. In the clear-cut area, herbaceous cover remained sparse 3 months after the start of the observation (0% in the skid trail and 15% in the clear-cut cypress forest). Meanwhile, in the clear-cut cedar forest, the cover rate increased by 100%, and the sediment yield was less than that for the unlogged forest plots. ^137^Cs activity concentration in the sediment was relatively low in the skid trail and clear-cut cypress; however, the maximum ^137^Cs yield was observed in the skid trail, where it was 3.6 to 21 times greater than the yields at the other plots. The skid trail also had an extremely high percentage of ^137^Cs transported (16 ± 8.5%), whereas the percentages did not exceed 1% in the clear-cut and unlogged forest plots.

**Table 2:** Properties of soils from the cores (10 cm in depth) and sediments from the plots.

Site	Soil^a^			Sediment				
	^137^Cs inventory (kBq m^−2^)	^137^Cs activity concentration (Bq kg^−1^)	Bulk density (g cm^−3^)	Amount of precipitation (mm)	Sediment yield (g m^−2^)	^137^Cs activity concentration (Bq kg^−1^)	^137^Cs yield (Bq m^−2^)	^137^Cs transported (%)^b^
**Clear-cut area**								
Skid trail	4.4 ± 1.9	44 ± 19	1.0 ± 0.18	1150	3300	180	580	16 ± 8.5
Clear-cut cedar forest	17 ± 7.5	340 ± 120	0.50 ± 0.063	1150	12	2200	28	0.19 ± 0.085
Clear-cut cypress forest	19 ± 3.1	520 ± 170	0.37 ± 0.054	1150	160	460	71	0.39 ± 0.062
**Forested area**								
Cedar forest	30 ± 15	970 ± 860	0.39 ± 0.14	960	27	2000	54	0.24 ± 0.16
Cypress forest	25 ± 12	580 ± 310	0.46 ± 0.13	960	51	3200	160	0.81 ± 0.43

^a^Mean ± standard deviation (n = 4).

^b^Means and standard deviations were calculated using ^137^Cs yield (n = 1) and ^137^Cs inventories (n = 4) obtained from soil samples.

### ^137^Cs export from the catchment through the stream system

#### Suspended solid fluxes during the pre- and post-clearcutting periods

**Fig 4:**
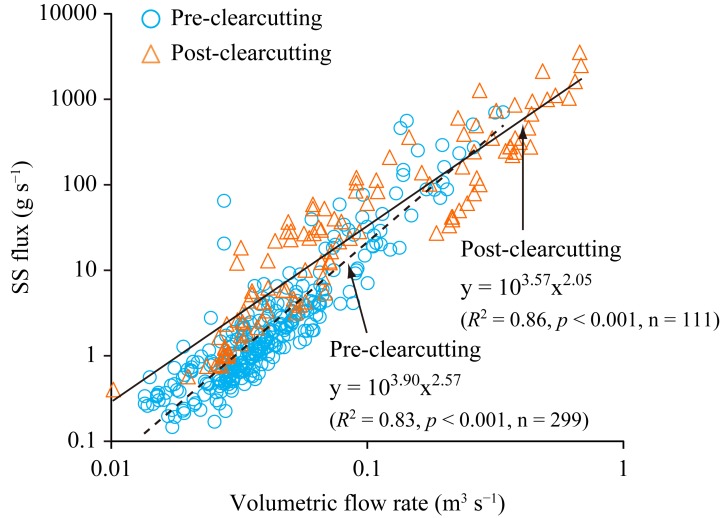
Comparison of measured suspended solids (SS) fluxes and volumetric flow rates in pre- and post-clearcutting periods.

A new LQ equation was established for the post-clearcutting period (*R*^2^ = 0.86, *p* < 0.001) from measured SS fluxes and volumetric flow rates at the outlet for the entire catchment (Fig 4). Regression coefficients of the two equations differed significantly based on a test of interaction effects (*p* < 0.001), and SS fluxes after clearcutting appeared to be higher when the volumetric flow rate was less than 0.1 m^3^ s^−1^.

**Fig 5:**
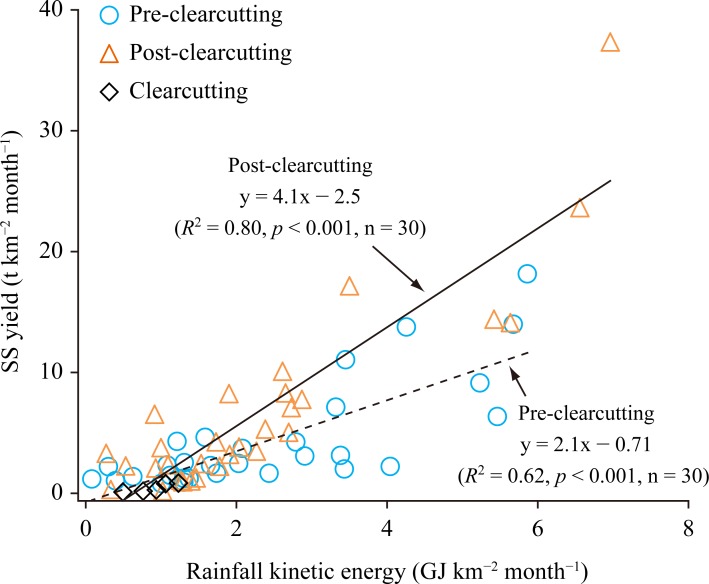
Comparison of monthly suspended solids (SS) yield and monthly rainfall kinetic energy in pre- and post-clearcutting periods.

In both the pre- and post-clearcutting periods, monthly SS flux per unit area (SS yield) strongly depended on monthly rainfall kinetic energy (Fig 5). The regression coefficients of these relational expressions differed significantly based on a test of interaction effects (*p* < 0.001). These results indicate that SS was more prone to export after clearcutting.

While both the pre- and post-clearcutting observation periods (2.5 years) had almost the same amounts of precipitation and rainfall kinetic energy (Table 3), SS flux during the post-clearcutting period was 1.5 times greater. SS flux estimated using turbidity was 0.55 times that calculated using the LQ equation in Fig 4, suggesting that the increase after clearcutting was not overestimated. Compared with the mean annual SS flux during the pre-clearcutting period, SS flux increased 1.4 times in the first year and 2.0 times in the second year after clearcutting. During the clearcutting period, the amount of precipitation was small, and the SS flux was very low (1.5 t). SS yield per unit rainfall kinetic energy (normalized SS yield) increased 1.6 times after the clearcutting.

**Table 3:** Precipitation, rainfall kinetic energy, and suspended solids flux during the study period.

Period	Amount of precipitation (mm)^a^	Rainfall kinetic energy (GJ km^−2^)	Suspended solids		
			Flux (t)	Yield (t km^−2^)	Normalized yield (t GJ^−1^)^b^
**Pre-clearcutting**					
May 2010 to Apr. 2011	1570	27	34	50	1.8
May 2011 to Apr. 2012	1700	30	31	46	1.6
May 2012 to Oct. 2012	890	15	24	35	2.3
**Total**	4160	73	89	131	1.8
**Clearcutting**					
Nov. 2012 to Mar. 2013	280	4.5	1.5	2.2	0.48
**Post-clearcutting**					
Apr. 2013 to Mar. 2014	1410	24	46	68	2.8
Apr. 2014 to Mar. 2015	1670	28	63	93	3.3
Apr. 2015 to Sep. 2015	950	16	28	41	2.5
**Total**	4030	69	137	203	3.0

^a^Values provided by the AMeDAS Kakioka station [39].

^b^Suspended solids yield normalized by rainfall kinetic energy.

**Fig 6:**
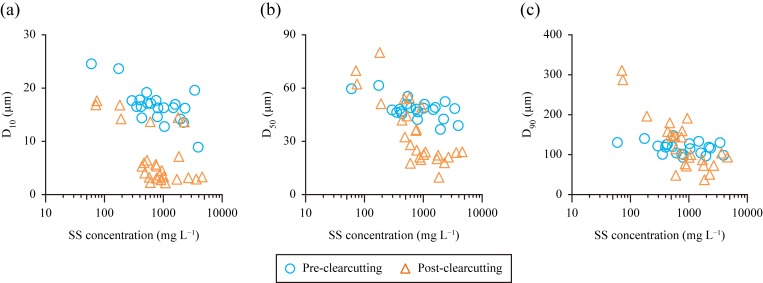
**Plots of suspended solid (SS) partitioned by relative particle size D_10_ (a), D_50_ (b), and D_90_ (c) against logarithmic SS concentration in the samples collected over the entire catchment outlet.** D_10_, D_50_, and D_90_ represent 10%, 50%, and 90% of the volume-based cumulative particle size distributions. Note that the *Y*-axis scales differ between (a), (b), and (c).

With respect to the particle size distribution of the SS, D_50_ and D_90_ in samples collected after clearcutting showed relatively wide ranges, and all relative particle sizes tended to be smaller as the SS concentration increased (Fig 6). This indicates a change in particle size distribution following clearcutting.

#### Temporal variation in the ^137^Cs activity concentration

**Fig 7:**
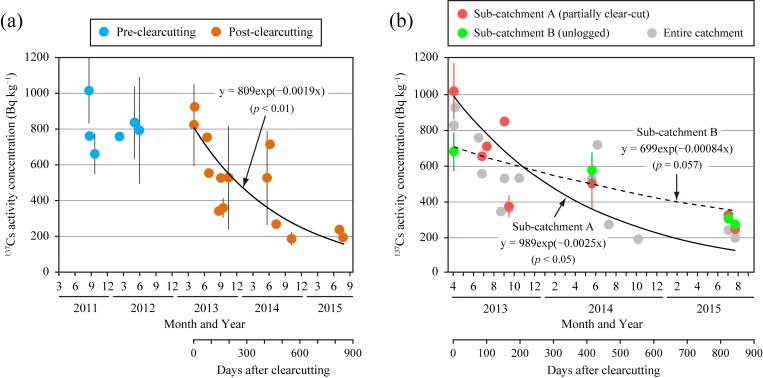
**Temporal variation in ^137^Cs activity concentrations in suspended solids at the outlets of (a) the entire catchment during the study period and (b) the sub-catchments and entire catchment during the post-clearcutting period.** The means (n = 1–7) of each rainfall event are plotted with the standard deviations (error bars).

During the pre-clearcutting period, ^137^Cs activity concentrations in the SS did not change at the outlet of the entire catchment (Fig 7A). The activity concentrations remained nearly constant until immediately following clearcutting; however, thereafter, the activity concentrations decreased with time, dropping to 24% from 3 to 844 days after clearcutting (820 ± 230 Bq kg^−1^ to 190 Bq kg^−1^). Based on the regression of the temporal decrease (Fig 7A), the effective half-life was determined to be 0.99 years (*p* < 0.05). A temporal decrease was also observed in the SS at the sub-catchment outlets. This decrease progressed faster in the outlet of sub-catchment A, although the number of sampling events at sub-catchment B may be not enough (Fig 7B). The activity concentrations declined to 24% and 40% from 3 to 844 days after clearcutting in sub-catchment A (1000 ± 160 Bq kg^−1^ to 240 Bq kg^−1^) and B (680 ± 110 Bq kg^−1^ to 270 Bq kg^−1^), respectively. The effective half-lives calculated from the regression equation in Fig 7B were 0.78 years in sub-catchment A (*p* < 0.05) and 2.26 years in sub-catchment B (*p* = 0.057).

#### Temporal variation in ^137^Cs flux

For the ^137^Cs flux calculation in the pre-clearcutting period, the mean of the activity concentration was used owing to the relative stability of the activity concentrations (Fig 7A). The flux before clearcutting was 24 MBq for the first year and 23 MBq for the second year (8 months), and the ^137^Cs export rates, the percentage of the flux in the total inventory of 9.0 GBq in the entire catchment, were 0.27% and 0.25%, respectively (Table 4). The export rate during the clearcutting period (5 months) was very low (0.014%) due to the relatively low precipitation (Table 3).

In the first year after clearcutting, the annual export rate was 0.30% (Table 4), which was slightly higher (1.1 times) than the value (0.27%) before clearcutting. The export rate, however, decreased below the value before clearcutting in the second year and was only 0.048% in the third year (half year). The export rate was 0.56% for 2.5 years following clearcutting, which was almost identical to the value (0.52%) for 1 year and 8 months before clearcutting.

**Table 4:** Flux and export rates of ^137^Cs during the study period.

Period	Flux (MBq)	Yield (MBq km^−2^)	Normalized yield (MBq GJ^−1^)^a^	Export rate (%)
**Pre-clearcutting**				
Mar. 2011 to Feb. 2012^b^	24	36	1.29	0.27
Mar. 2012 to Oct. 2012	23	34	1.76	0.25
**Total**	47	70	1.48	0.52
**Clearcutting**				
Nov. 2012 to Mar. 2013	1.2	1.8	0.41	0.014
**Post-clearcutting**				
Apr. 2013 to Mar. 2014	27	41	1.69	0.30
Apr. 2014 to Mar. 2015	19	28	1.00	0.21
Apr. 2015 to Sep. 2015	4.4	6.5	0.40	0.048
**Total**	51	75		0.56

^a137^Cs yield normalized by rainfall kinetic energy.

^b^The starting date was the deposition date (March 16, 2011) of FDNPP-derived radiocesium at the study site [49].

Before clearcutting, ^137^Cs yield per unit rainfall kinetic energy (normalized ^137^Cs yield) was 1.48 MBq GJ^−1^ on average (Table 4). After clearcutting, the normalized yield increased slightly to 1.69 MBq GJ^−1^ in the first year and decreased to 0.40–1.00 MBq GJ^−1^ in the second and third years.

## Discussion

### Factors controlling the increase in ^137^Cs export

The annual SS yield increased 1.4–2.0 times following clearcutting (Table 3), whereas the ^137^Cs yield hardly increased (Table 4). This result is consistent with the results of Wallbrink et al. [32], who showed limited ^137^Cs loss in a clear-cut catchment in Australia (see Introduction). These findings oppose the assumption that ^137^Cs export is increased by clearcutting. However, to predict export in other catchments, it is necessary to understand why the increase in ^137^Cs export was curbed.

Two factors could control the increase in ^137^Cs export in the catchment in this study. First, the increase in SS yield following clearcutting was relatively small (less than double; Table 3) compared with those in temperate forests worldwide (0.2–150 times with a median of 4.7 times following logging [30]).

The second factor is the rapid decrease in the ^137^Cs activity concentration in the SS (Fig 7A). The effective half-life observed at the entire catchment outlet (0.99 years) was considerably shorter than the physical half-life (30.2 years) and was closer to that in the partially clear-cut sub-catchment A (0.78 years) than to that in the unlogged sub-catchment B (2.26 years) (Fig 7B). Consequently, the rapid decrease in the activity concentration in the entire catchment was likely strongly affected by changes in sub-catchment A. The decrease in sub-catchment B can be attributed to natural attenuation possibly caused by the erosion of subsurface material depleted in ^137^Cs and the temporal decline in finer materials with relatively high ^137^Cs activity concentrations in preferential erosion sites. These suggest that both clearcutting and natural attenuation were involved in the rapid decrease in sub-catchment A. It is necessary to identify potential sources of ^137^Cs and SS exported from the clear-cut area to understand why clearcutting decreased the activity concentration in SS.

### Potential sources of ^137^Cs and SS exported from the clear-cut area

Four potential sources of the increase in ^137^Cs and/or SS export in the clear-cut area were as follows: the skid trails, clear-cut forest floors with scarce understory vegetation, slope failure, and stream channels.

#### Skid trails

The skid trail plot showed the highest sensitivity to soil erosion (Table 2). This was probably due to the absence of an organic horizon and undergrowth (Table 1), which intensifies soil detachment by raindrops, and because soil compaction with traffic (Table 2) leads to low permeability of the soil and subsequent high levels of infiltration-excess overland flow [36,37,50]. Sediments from skid trails may easily flow into streams via road-stream crossings without sediment mitigation strategies [35], and such crossings were found in the clear-cut area (see Materials and Methods). The skid trails therefore were a likely source of the increasing SS export. Infiltration-excess overland flow from disturbed areas, such as skid trails, may contribute substantially to changes in peak discharge and flow volume during small storms [51]. The SS flux at the catchment outlet increased at the time of low to medium volumetric flow rates after clearcutting (Fig 4), suggesting that the skid trails contributed to the SS flux increase in small to medium storms.

The skid trails were also a potential source of increased ^137^Cs export based on the 3.6–21 times greater ^137^Cs yield than those in the other plots (Table 2). Meanwhile, the activity concentration in the sediment was lower than those in the other plots (Table 2) and in the SS in sub-catchment A during the experimental period (Fig 7B). This is likely because the surface soil of the skid trails included subsoil layers with low ^137^Cs content (Fig 3), since they were constructed by cutting and filling slopes (see Materials and Methods). Many studies have reported that the mixing of subsurface materials into SS dilutes the activity concentration of ^137^Cs derived from nuclear weapons testing (e.g., [52–54]). These conditions strongly suggest that the skid trails contribute to the decrease in the activity concentration in SS.

#### Clear-cut forest floors with sparse undergrowth

The clear-cut cypress forest plot showed higher sensitivity to soil erosion than that of other three forests plots (Table 2), likely due to the sparse herbaceous cover (15%). However, the percentage of ^137^Cs transported was comparable to those in the three plots because the activity concentration was relatively low in the sediment (Table 2). This activity concentration of 460 Bq kg^−1^ was between the values observed in the organics and the A horizons in the site (Fig 3B), implying a role of the erosion of the A horizon, to some extent. In contrast, the activity concentration in other forests plots (>2000 Bq kg^−1^, Table 2) was close to those in the organic horizons and the top of A horizon in the sites (Fig 3B), indicating that erosion occurred largely in the organic horizons. Numerous ruts were made in the clear-cut forest floor during felling and trampling, and ruts in the A horizon of the clear-cut cypress forest plot may collapse due to sparse undergrowth and weak soil-binding by roots. Thus, clear-cut forest floors with little undergrowth were not a potential source of increased ^137^Cs export and could contribute to the decreased activity concentration in the SS.

#### Slope failure and stream channels

Slope failure, which occurred beside a stream (Fig 1), may contribute to the increased ^137^Cs and SS export. Most of the collapsed soil, however, is likely subsoil sheltered from ^137^Cs fallout, and the slope failure perhaps affected the decrease of the activity concentration in the SS.

Clearcutting may increase peak discharges of streams due to changes in hydrological processes, including decreased evapotranspiration and increased overland flow from disturbed surfaces [51,55]. Increased peak discharges may lead to accelerated erosion of stream banks and beds. The particle size of the channel bank/bed material is typically coarser than that of top soil material (e.g., [56]), and the channel bed consists of sand and gravel in the study site (see Materials and Methods). However, SS was smaller in size after clearcutting for high SS concentrations (Fig 6). This suggests that after clearcutting, substantial SS was generated not from the channel bank/bed but from potential SS sources with topsoil (i.e., skid trails, clear-cut forest floors with sparse undergrowth, and slope failure). Although part of the streambanks might retain the initial fallout ^137^Cs, streambank erosion probably did not contribute substantially to the increased ^137^Cs export. Similarly, streambeds were not likely to be a major ^137^Cs source because most of the initial fallout ^137^Cs in streambeds presumably flowed out before clearcutting.

For the reasons stated above, we conclude that the increased SS export after clearcutting was probably due to the erosion of skid trails and clear-cut forest floors with sparse undergrowth as well as slope failure, whereas the low activity concentrations in the sediments dropped the concentration in the SS, thereby controlling the increase in ^137^Cs export.

### Net ^137^Cs export increased by clearcutting

Since natural attenuation likely also contributed to the decrease in the ^137^Cs activity concentration following clearcutting, the net ^137^Cs export increased by clearcutting remains unclear. We estimated undisturbed ^137^Cs flux assuming that the catchment was not clear-cut and compared the undisturbed flux to the actual flux, taking the difference as the net flux increased by clearcutting. First, the undisturbed SS fluxes at 10-min intervals were calculated as follows:

$$SSF_{nc}=SSF_{post}/\left(NY_{post}/NY_{pre}\right)$$ (4)

where *SSF* is the SS flux (t s^−1^), *NY* is the normalized SS yield (t GJ^−1^), the subscript *nc* represents a value based on the assumption that the catchment was not clear-cut, the subscript *post* refers to a value in the first, second, or third year after clearcutting, and the subscript *pre* indicates the mean before clearcutting. Then, the undisturbed activity concentration in the SS was calculated using Eq (1) and a rate of decrease of 60% at 844 days observed at the outlet of sub-catchment B. The equation is represented as follows:

$$C_{nl}=809\;exp\left(-0.0011\;D\right)$$ (5)

where the *y*-intercept (809) is from the regression expression for the entire catchment (Fig 7A), and *D* is the number of days after clearcutting (see Eq 1). The undisturbed ^137^Cs flux at 10-min intervals was calculated by multiplying *SSF*_*nc*_ in Eq (4) and *C*_*nl*_ in Eq (5).

**Fig 8:**
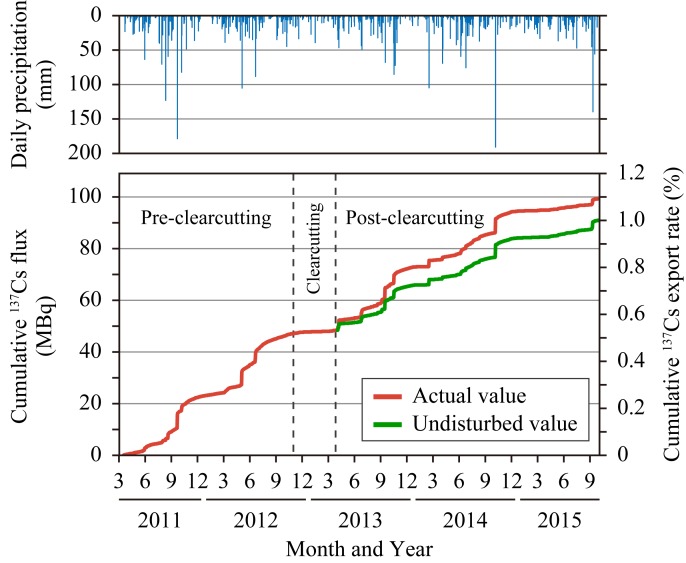
Cumulative flux and export rate of ^137^Cs and daily precipitation from the initial deposition (March 16, 2011, [49]). Actual values represent measured values. Undisturbed values were calculated under the assumption that the catchment was not clear-cut.

The undisturbed flux from the initial deposition to September in 2015 was estimated to be 91 MBq, corresponding to 1.01% of the export rate (Fig 8), while the actual values were 99 MBq and 1.10%. Thus, the net increases for 2.5 years were 8.4 MBq and 0.092%, and the export increased by 1.2 times due to clearcutting. After clearcutting, unlike in the SS flux, the net increase of ^137^Cs flux decreased year by year, and the actual flux was less than the undisturbed value in the third year (S2 Table). This may be because most of the ^137^Cs sorbed by soil sensitive to erosion was removed from the clear-cut area through export after clearcutting. These results imply that the impact of clearcutting on ^137^Cs export was temporary in this catchment.

Although this is one of the few valuable studies to reveal an effect of artificial disturbance on radiocesium export from a forested catchment, the results are derived from a case study. The impacts of clearcutting on trace element discharge to streams are known to vary widely due to both environmental and operational differences [31,57]. Additional research considering different conditions is necessary to understand general trends in radiocesium export and to identify major sources for developing suitable management strategies to limit the export.

## Conclusions

We aimed to determine the impact of clearcutting on ^137^Cs export and investigated changes in the export over time following the clearcutting of 13% of a Japanese forested catchment affected by the Fukushima nuclear accident. While annual SS export increased 1.4–2.0 times after clearcutting, ^137^Cs export hardly increased (up to 1.1 times). This can be attributed to a rapid decrease of ^137^Cs activity concentration in SS after clearcutting. This decrease was likely a result of both natural attenuation and SS derived from sources with a low activity concentration in the clear-cut area. Even the net ^137^Cs export increased by clearcutting was only 0.092% of the inventory in the catchment for 2.5 years. Monitoring sediment from the hillslopes suggests that the skid trail was rather sensitive to soil erosion and a source of the increased ^137^Cs export. A slope failure in the clear-cut area was also a potential source. To develop suitable management strategies for limiting export, further research under different environmental and operational conditions is required to determine general export trends and major sources.

## Supporting information

S1 Fig Observation periods of sediment transferred on hillslopes and exports of suspended solids (SS) and ^137^Cs from the catchment. (EPS)

S1 Table Physical properties, total contents of C and N, and ^137^Cs distributions in soil profiles (20 cm in depth). (XLSX)

S2 Table Suspended solids flux, ^137^Cs flux, and ^137^Cs export rate (assuming no clearcutting in the catchment). (XLSX)
